# Choosing the safest acute combination therapy during prophylactic treatment: pharmacokinetic and pharmacodynamic considerations

**DOI:** 10.1186/1129-2377-16-S1-A36

**Published:** 2015-09-28

**Authors:** Luana Lionetto, Paolo Martelletti

**Affiliations:** Advanced Molecular Diagnostics Unit, Sant'Andrea Hospital, Rome, Italy; Department of Clinical and Molecular Medicine, Sapienza University of Rome, Rome, Italy; Regional Referral Headache Centre, Sant'Andrea Hospital, Rome, Italy

Drugs used in the treatment of migraine have been recently reported to be highly associated with the occurrence of clinically significant drug-drug interactions (DDIs). The multiple drug therapy regimen is widely used for migraine treatment, particularly for chronic migraine. In fact, additional pharmacological agents are usually administered during an acute migraine attack in patient chronically treated with prophylactic therapy. The wide variety of drugs available for migraine prophylactic and acute treatment, and consequently their pharmacological interactions, might complicate the choice of a safe combination therapy. The most frequently used drugs for the prophylactic therapy of migraine belong to the antiepileptic, β-blockers, tricyclic, SSRIs and SNRIs antidepressants and antihistamine medications while acute migraine attacks are treated with triptans, NSAIDs and ergot derivatives. Moreover, in the last few years several of the latter drugs have been combined in new formulations for clinical use in order to improve treatment efficacy and, consequently, the compliance of the patient. Drug-drug interactions might occur at receptors level, both in the Central Nervous System (CNS) and in the periphery, at the major metabolic pathways levels (i.e., CYP450 enzymes) and at the protein binding level. One of the most widely known examples of the severity of such interactions is represented by the serotoninergic syndrome induced by the co-administration of serotoninergic antidepressants and triptans.

In the management of chronic migraine, usually prophylactic treatment is already administered, therefore the choice of an additional drug for the acute attack should be decided considering the specific DDIs.

Therefore, the aim of this study was to schematically discuss the prophylactic-acute drug-drug interactions from a pharmacokinetic and pharmacodynamic point of view.

